# βVPE is involved in tapetal degradation and pollen development by activating proprotease maturation in *Arabidopsis thaliana*

**DOI:** 10.1093/jxb/erz560

**Published:** 2019-12-20

**Authors:** Ziyi Cheng, Xiaorui Guo, Jiaxue Zhang, Yadi Liu, Bing Wang, Hui Li, Hai Lu

**Affiliations:** 1 Beijing Advanced Innovation Center for Tree Breeding by Molecular Design, Beijing Forestry University, Beijing, China; 2 College of Biological Sciences and Biotechnology, Beijing Forestry University, Beijing, China; 3 Shanghai Jiao Tong University, China

**Keywords:** Cysteine protease maturation, pollen grain, tapetum, vacuole, βVPE

## Abstract

Vacuolar processing enzyme (VPE) is responsible for the maturation and activation of vacuolar proteins in plants. We found that *βVPE* was involved in tapetal degradation and pollen development by transforming proproteases into mature protease in *Arabidopsis thaliana*. *βVPE* was expressed specifically in the tapetum from stages 5 to 8 of anther development. The βVPE protein first appeared as a proenzyme and was transformed into the mature enzyme before stages 7–8. The recombinant βVPE protein self-cleaved and transformed into a 27 kDa mature protein at pH 5.2. The mature βVPE protein could induce the maturation of CEP1 *in vitro*. *βvpe* mutants exhibited delayed vacuolar degradation and decreased pollen fertility. The maturation of CEP1, RD19A, and RD19C was seriously inhibited in *βvpe* mutants. Our results indicate that βVPE is a crucial processing enzyme that directly participates in the maturation of cysteine proteases before vacuolar degradation, and is indirectly involved in pollen development and tapetal cell degradation.

## Introduction

Tapetal cells are degraded through programmed cell death (PCD) to provide various nutrients for pollen development, particularly the formation of pollen exine. Premature or abrogated PCD of tapetal cells can disrupt the supply of these nutrients to microspores, resulting in sterile pollen (Ku *et al.*, 2003; Varnier *et al.*, 2005). Many genes, including those encoding transcription factors and functional proteins, such as *Arabidopsis thaliana* DYSFUNCTIONAL TAPETUM1 (DYT1) (Feng *et al.*, 2012), DEFECTIVE IN TAPETAL DEVELOPMENT AND FUNCTION1 (TDF1) (Zhu *et al.*, 2008), MON1/CCZ1 (Cui *et al.*, 2017), and MYB80 (formerly MYB103) (Phan *et al.*, 2012), and rice (*Oryza sativa*) TAPETUM DEGENERATION RETARDATION (TDR) (Li *et al.*, 2006), EAT1 (Ono *et al.*, 2018), and PERSISTENT TAPETAL CELL1 (PTC1) (Li *et al.*, 2011), are associated with tapetal PCD (Lin *et al.*, 2017). Many cysteine protease enzymes are ubiquitously involved in tapetal cell degeneration and pollen development (Song *et al.*, 2016). For example, OsCP1, a rice cysteine protease, plays an important role in pollen development and is regulated by TDR (Lee *et al.*, 2004; Li *et al.*, 2006). BnMs3 participates in tapetum development, microspore release, and pollen wall formation in *Brassica napus* (Zhou *et al.*, 2012). The VPE-like protease NtTPE8 is expressed in the integumentary tapetum and is involved in seed development (Wang *et al.*, 2019). *Arachis diogoi* cysteine protease (AdCP) expressed under the TA-29 promoter induced complete male sterility in Indian mustard, *Brassica juncea* (Gautam *et al.*, 2019). Targeted expression of a cysteine protease inhibitor, from a wild peanut, restores fertility in cysteine protease-induced male-sterile tobacco plants (Shukla *et al.*, 2016). Previous studies in our lab revealed that CEP1 plays an irreplaceable executor role during tapetal PCD and affects pollen development (Zhang *et al.*, 2014). A vacuolar system mediated by vacuolar processing enzyme (VPE) is considered a cellular suicide strategy in plant development and cell death programs (Tang *et al.*, 2016; Teper-Bamnolker *et al.*, 2017). VPE mediates the initial activation of some other vacuolar enzymes, which then degrade the vacuolar membrane and initiate the proteolytic cascade leading to PCD (Yamada *et al.*, 1999; Lam, 2005; Hatsugai *et al.*, 2015; Gong *et al.*, 2018). VPE is also capable of processing several seed proteins, including the 2S albumins and 11S globulins (Shimada *et al.*, 2003).

VPEs, which are asparagine-specific cysteine proteinases exclusively located in the vacuoles of plants, are synthesized as larger, inactive proprotein precursors, from which the C- and N-terminal propeptides are sequentially removed in acidic conditions (pH 5.5) via self-catalysis to produce the active mature forms (Kuroyanagi *et al.*, 2002; Misas-Villamil *et al.*, 2013). The Arabidopsis genome contains four VPE genes (αVPE, βVPE, γVPE, and δVPE), which can be separated into two subfamilies: the vegetative-type αVPE and γVPE, and the seed-type βVPE and δVPE (Kinoshita *et al.*, 1999; Gruis *et al.*, 2002; Greenberg and Yao, 2004). Previous research into plant VPEs has mostly focused on plant senescence, terminal differentiation, and pathogen-induced hypersensitive cell death. αVPE and γVPE, which are up-regulated during wounding, senescence, and pathogen infection, may play vital roles in plant cell death (Kinoshita *et al.*, 1999; Yamada *et al.*, 2004). Promoter–β-glucuronidase (GUS) analyses revealed the up-regulation of αVPE in dying cortex cells located next to the emerging lateral root (Kinoshita *et al.*, 1999) and of γVPE in dying circular cell clusters of anthers during the later stages of pollen development (Hatsugai, 2015). In contrast, βVPE is essential for storage protein processing (Shimada *et al.*, 2003), and δVPE, which is specifically expressed in the seed coat, is associated with cell death (Nakaune *et al.*, 2005). OsVPE1, which is a homolog of Arabidopsis βVPE, is a cysteine protease that plays a crucial role in the maturation of rice glutelins (Wang *et al.*, 2009). Additionally, abnormal accumulation of the precursors of 12S globulins has been reported in Arabidopsis mutants lacking VPE (Gruis *et al.*, 2002; Shimada *et al.*, 2003).

Previous studies have found that *βVPE* expression is significantly down-regulated in *spl/nzz* and *ems1/exs* Arabidopsis mutants, which display dramatically altered anther cell differentiation patterns (Wijeratne *et al.*, 2007). In particular, expression of βVPE is detected in the stamen, flower pedicel, pollen, petal, carpel, and sepal during flower development, as well as in seeds and root tips (Kinoshita *et al.*, 1999). βVPE is expressed in roots, flowers, buds, and ovules, and is specifically expressed during ovule development in *Vitis vinifera* (Tang *et al.*, 2016). For these reasons, βVPE is speculated to play an essential role in flower development, but its exact function and corresponding mechanism of action remain uncertain.

To investigate the role of βVPE in anther development, we characterized the expression of *βVPE* in the anther and the phenotype of the *βvpe* mutant. We also detected the maturation of the cysteine proteinases CEP1, RD19A, and RD19C by βVPE *in vitro* and *in vivo*. Our results indicate that βVPE acts as a trigger during anther development by activating cysteine proteinases in acidic vacuolar environments. Here, we present the first evidence of the activation of papain-like cysteine proteases by VPE during anther development in *A. thaliana*.

## Materials and methods

### Plant materials and growth conditions

*Arabidopsis thaliana* Columbia was used as the wild-type control. Plants were grown in a soil mixture (3:1:1 mixture of peat moss-enriched soil:vermiculite:perlite) with a 14 h light/10 h dark photoperiod at 23 °C. Homozygous T-DNA insertion mutants were identified by PCR using βVPE-BP/LP/RP primers. CS_1007412: BP, 5'-ATTTTGCCGATTTCGGAAC-3'; LP, 5'-TGACCAATTCCACAAACTTCC-3'; RP, 5'-TGTCGGCATAAGAATCTTTGG-3'; and SAIL_50_F12: BP, 5'-TCAAACAGGATTTTCGCCTGCT-3'; LP, 5'-TGACCAATTCCACAAACTTCC-3'; RP, 5'-TGTCGGCATAAGAATCTTTGG-3'.

### Characterization of the mutant phenotype

Arabidopsis plants were photographed using a digital camera (Coolpix 9100; Nikon, Tokyo, Japan). Arabidopsis pollen germination images were acquired using an M165 C microscope (Leica, Wetzlar, Germany). To evaluate the viability of mature pollen grains, germination was assessed by culturing fresh pollen grains in germination medium (pH 5.8) containing 3 mM CaCl_2_, 1 mM H_3_BO_3_, 56 mM inositol, 1% (w/v) agar, and 15% (w/v) sucrose at 25 °C for 3 h. For each group, 200 pollen grains were counted. Each experiment was repeated three times with both mutants and wild-type plants.

### Semi-thin sections

Freshly dehisced anthers were collected at stages 8–13 from both wild-type and mutant plants, and fixed in glutaraldehyde fixing solution [2.5% glutaraldehyde, 0.1 M phosphate-buffered slaine (PBS), pH 7.4] for 4 h (Zhang *et al.*, 2014). The samples were then pre-stained with Sorensen buffer overnight at room temperature before being dehydrated in an alcohol gradient series (3 h each at 50, 70, 95, and 100% alcohol) and embedded in LR White at 60 °C for 24 h. Semi-thin sections of 2 mm were cut using a UC6 ultramicrotome (Leica), stained with 1% toluidine blue O (Sigma-Aldrich), and photographed using a Leica DM 6000 B microscope.

### Scanning electron microscopy

Pollen grains collected from freshly dehisced anthers of both wild-type and mutant plants were mounted on SEM stubs. The mounted samples were coated with palladium–gold in a sputter coater (E-1010; Hitachi, Tokyo, Japan) and examined by SEM (S-3400N; Hitachi) at an acceleration voltage of 10 kV. For each line, pollen grains from six independent plants were counted for SEM.

### Transmission electron microscopy

Both wild-type and mutant anthers at stages 9–12 were pre-fixed and embedded (Zhang *et al.*, 2014). Ultrathin sections (70 nm) were obtained with a UC6 ultramicrotome (Leica) and double-stained with 2% (w/v) uranyl acetate and 2.6% (w/v) lead citrate aqueous solution. Observations and image capture were performed with an H-7650 transmission electron microscope (Hitachi) at 80 kV and an 832 charge-coupled device camera (Gatan, Inc., Pleasanton, CA, USA).

### Tunel

Freshly dehisced anthers were collected at stages 8–13 from both wild-type and mutant plants and fixed in polyoxymethylene and glutaraldehyde fixing solution (4% polyoxymethylene, 2.5% glutaraldehyde, 0.1 M PBS, pH 7.4) for 4 h. The samples were then pre-stained with 1% safranin overnight at room temperature before being dehydrated in an alcohol gradient series (1 h each at 70, 85, 90, and 100% alcohol) and cleared in a xylene/alcohol gradient series (1 h each at 70, 85, 90, and 100% xylene). The samples were incubated in xylene/paraffin (1:1) overnight at 38 °C and dipped in 58 °C paraffin three times (1 h per incubation). The 2 mm Paraffin sections of treated buds were assessed with a TUNEL (terminal deoxynucleotidyl transferase dUTP nick end labeling) apoptosis detection kit [fluorescein isothiocyanate (FITC)] according to the supplier’s instructions. TUNEL-positive nuclei are labeled by FITC-12-dUTP. Samples were observed at 520 nm using a Leica DMI6000 CS confocal laser scanning microscope.

### Molecular cloning and plasmid construction

A 1847 bp promoter of *βVPE* (Pro*βVPE*) was amplified with Pro-βVPE-F/R primers (F: 5'-ATAAGTAGTAATATCAAGTTC-3'; R: 5'-CAATTGTCTAATTATATTTAAT-3'). The promoter was cloned into the pCAMBIA 1300 vector for *Proβvpe:GUS* fusion construct and transformed into Arabidopsis. The ORF minus the first 63 bp of *βVPE* cDNA was amplified by PCR with the two *βVPE*-CM-F/R primers (F: 5'-GAGTCACGCGGTCGGTTCGAG-3'; R: 5'-TCAGGCGCTATAGCCTAAG-3') and inserted downstream of the pET30a plasmid T7 promoter (Novagen). The expression, extraction, purification, and renaturation of the CEP1 and βVPE proteins were performed according to the procedure described by Zhang *et al.* (2014).

### qRT–PCR analyses

*βVPE* expression in different Arabidopsis tissues and buds of different stages was assessed by quantitative reverse transcription–PCR (qRT–PCR) using SYBR Green qPCR mix (LabAid; Thermo Scientific) on an iQ5 Multicolor Real-Time PCR detection system (Bio-Rad) using the qRT-*βvpe*-F/R primers (F: 5'-GATTCTTATGCCGACAGAGG-3'; R: 5'-CCTGGTGTCTGTAGTTTCCA-3'). The PCR conditions were as follows: 94 °C for 3 min; 40 cycles at 94 °C for 10 s, 55 °C for 20 s, 72 °C for 20 s, and 60 °C for 30 s; and 72 °C for 1 min. The PCR product is 116 bp (from base pair 215 to 331 downstream of ATG). To normalize expression data, qRT-actin-F/R (F: 5'-CGTATGAGCAAGGAGATCAC-3'; R: 5'-CACATCTGTTGGAAGGTGCT-3') was used as an internal control. Data were analyzed using the iQ5 (Bio-Rad) software, and differences in gene expression were calculated using the 2–DDCt analysis method.

### Immunoblotting

Anti-mature βVPE antibody at a 1:200 dilution followed by affinity-purified goat anti-rabbit IgG horseradish peroxidase (HRP)-conjugated antibody (CW Bio) at a 1:4000 dilution were used for immunoblotting. ECL Plus protein gel blotting detection reagents (CW Bio) were used as the HRP substrate and were exposed in the Fusion X7 chemiluminescence imaging system.

### GUS staining assay

Flower buds at different stages from heterozygous transgenic lines were treated with 90% (v/v) acetone for 1 h on ice. The tissues were subsequently stained with X-Gluc solution and incubated at 37 °C for 12 h to visualize GUS activity. The samples were first cleared with 75% (v/v) ethanol and fixed in formaldehyde–acetic acid–ethanol (FAA) for 4 h. The samples were then pre-stained with 1% safranin overnight at room temperature before being dehydrated in an alcohol gradient series (1 h each at 70, 85, 90, and 100% alcohol) and cleared in a xylene/alcohol gradient series (1 h each at 70, 85, 90, and 100% xylene). The samples were incubated in xylene/paraffin (1:1) overnight at 38 °C and dipped in 58 °C paraffin three times (1 h per incubation). Paraffin-embedded samples were sectioned according to Zhang *et al.* (2014) and observed under an M165 C microscope.

### Accession numbers

Sequence data from this study can be found in the Arabidopsis Genome Initiative database under accession numbers AT1G62710 (*βVPE*), AT5G50260 (*CEP1*), AT4G39090 (*RD19A*), and AT4G16190 (*RD19C*).

## Results

### Characterization of *βVPE* expression in Arabidopsis

**Fig. 1.**
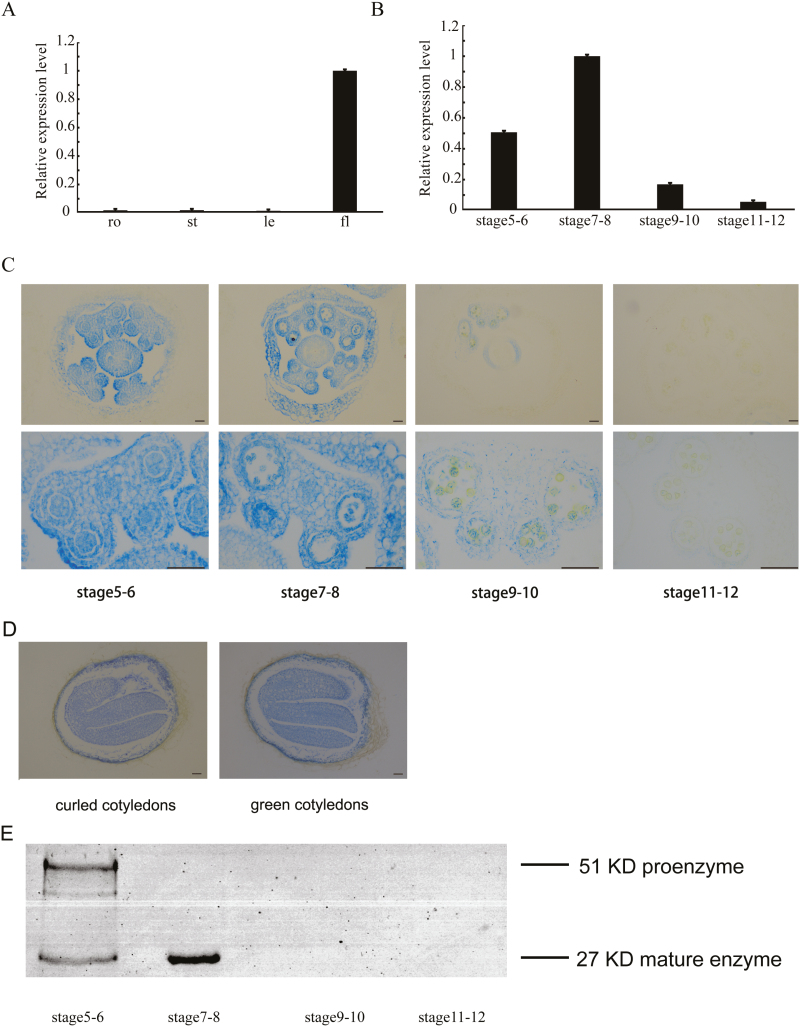
*βVPE* expression pattern. (A) *βVPE* spatial and temporal expression analyses performed by qRT–PCR. Fl, flower; le, leaf; ro, root; st, stem. (B) qRT–PCR of *βVPE* expression in wild-type bud tissues at different developmental stages. Bars represent SDs. The expression of *βVPE* in stages 7–8 was selected as 1. (C) Histochemical assay for GUS activity in anthers during stages 5–12. Scale bar=20 μm. (D) GUS activity in the developing seeds during the curled cotyledons stage to the green cotyledons stage. Scale bar=20 μm. (E) Immunoblot analysis of total anther protein extracts from stages 5–12 with anti-mature *βVPE* antibody.

To identify the function of *βVPE* (AT1G62710) during Arabidopsis anther development, we investigated *βVPE* expression characteristics. We performed qRT–PCR analysis with total RNA extracted from various organs, including roots, stems, leaves, and buds. *βVPE* was highly expressed in flower buds but almost undetectable in roots, stems, and leaves (Fig. 1A). The development of Arabidopsis anthers is divided into 14 stages based on morphological landmarks that correspond to cellular events visible under a microscope (Sanders *et al.*, 1999). The expression of *βVPE* appeared in stages 5–6, reached its maximum level in stages 7–8, and then declined sharply to a barely detectable level in stages 9–12 (Fig. 1B). An 1847 bp promoter of *βVPE* (Pro*βVPE*) was cloned into the pCAMBIA 1300 vector for the *Proβvpe:GUS* fusion construct and transformed into Arabidopsis. GUS activity was detected in the bud, including sepal, petal, anther, and pistil during stages 5–8, and greatly decreased during stages 9–12 (Fig. 1C). GUS activity was detected in the epidermis, endothecium, middle layer, and tapetum of the anther during stages 5–8, declined sharply in stages 9–10, and was almost undetectable in stages 11–12 (Fig. 1C). GUS activity was also detected in the late developing seeds during the curled cotyledons to green cotyledons stage (Fig. 1D). Taken together, these results indicate that the *βVPE* gene is expressed abundantly in Arabidopsis anther development from stages 5 to 8.

We performed immunoblotting using an antimature-βVPE antibody in anthers from stages 5–12 to evaluate βVPE maturation time. The results revealed that the 51 kDa proenzyme was detected only in stages 5–6, while the 27 kDa mature enzyme appeared during stages 5–8. However, during stages 9–12, the quantity of the 51 kDa proenzyme and the 27 kDa mature enzyme greatly decreased, becoming barely detectable (Fig. 1E).

### Morphology of *βvpe* mutants

**Fig. 2.**
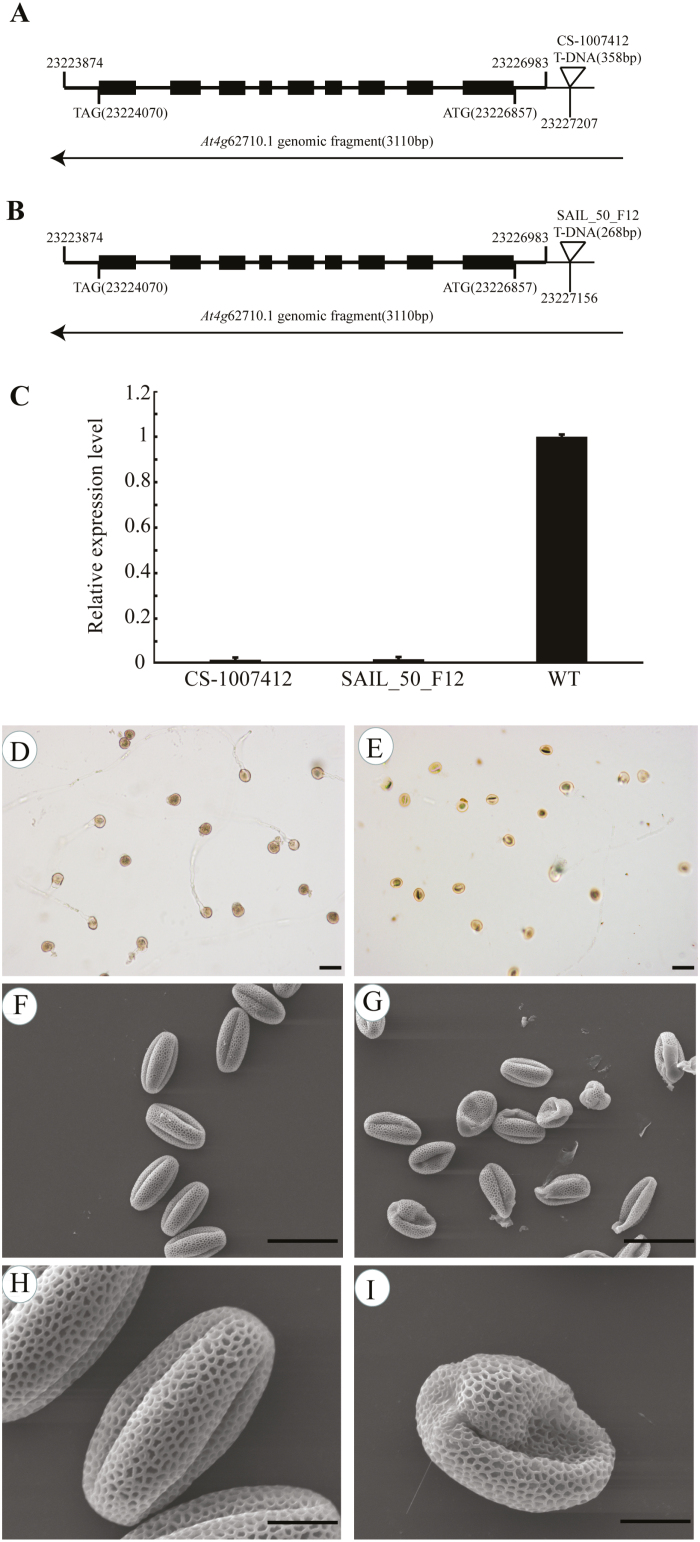
The phenotype of Arabidopsis *βvpe* mutant plants. (A) CS_1007412 T-DNA insertion positions in *At1g62710.1*. (B) SAIL_50_F12 T-DNA insertion positions in *At1g62710.1*. (C) *βVPE* expression analyses in mutants. (D) Germination rate of wild-type pollen. (E) Germination rate of *βvpe* mutant pollen. (F) and (H) SEM of wild-type pollen. (G) and (I) SEM of *βvpe* mutant pollen. (D–G) scale bar=50 μm; (H) and (I) scale bar=10 μm.

To identify the function of *βVPE* during Arabidopsis anther development, we obtained two T-DNA insertion mutants (CS_1007412 and SAIL_50_F12) from the Arabidopsis Biological Resource Center (ABRC). The position where the 358 bp T-DNA fragment inserted in CS_1007412 is 224 bp upstream of the transcription start site, and the position where the 268 bp T-DNA fragment inserted is 173 bp upstream of the transcription start site (Fig. 2A, B). Homozygous T-DNA insertion mutants were identified by PCR using βVPE-BP/LP/RP primers. The results of qRT–PCR (Fig. 2C) revealed that the expression of *βVPE* was completely suppressed in the mutants. The Arabidopsis *ACTIN1* gene (AT2G37620) was used as the reference for normalization. CS_1007412 was used for further analysis and is referred to as *βvpe-1*. *βvpe-1* mutant plants displayed a normal (wild-type) phenotype during vegetative and early generative development stages, but showed markedly impaired pollen development resulting in sterile pollen grains with abnormal pollen morphology. The germination rate of pollen grains *in vitro* was significantly lower in *βvpe-1* (45.13%, 116 of 257) than in the wild type (85.54%, 219 of 256; Fig. 2D, E). An SEM examination revealed that mature pollen grains in wild-type plants (82.14%, 92 of 112) were uniformly spheroid and had finely reticulate ornamentation on their surfaces (Fig. 2F, H), while the *βvpe-1* mutant produced some mature pollen grains that were similar to the wild type and some abnormal pollen grains (50.00%, 56 of 112) exhibiting shrunken, collapsed, and gemmate–baculate sculpture without regularly reticulate ornamentation (Fig. 2G, I). Six plants of each line were examined.

### Anther development in *βvpe* mutants

**Fig. 3.**
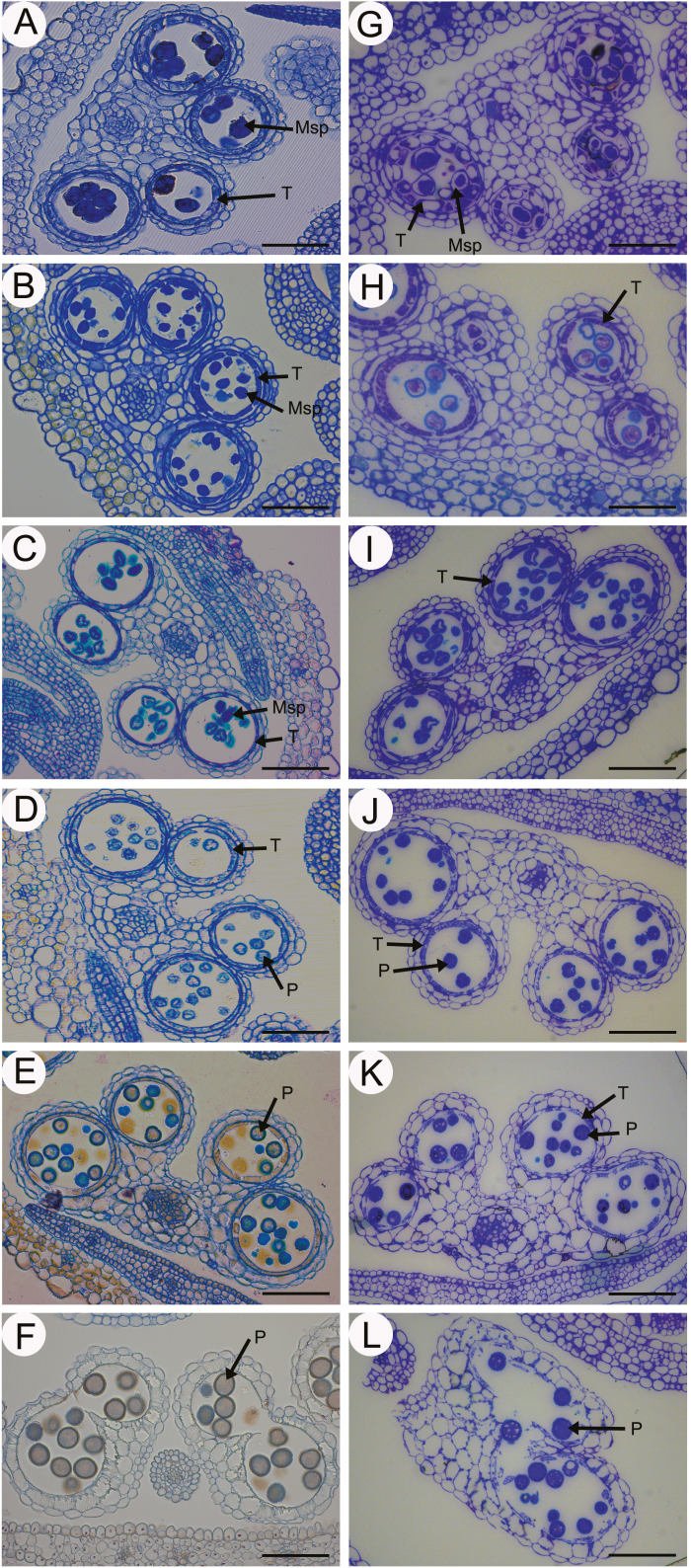
Anther development in the wild type and *βvpe* mutant. The wild type (A–F) and *βVPE* mutant (CS_1007412) (G–L) during anther development. (A) and (G) stage 7; (B) and (H) stage 8; (C) and (I) stage 9; (D) and (J) stage 10; (E) and (K) stage 11; (F) and (L) stage 12. Scale bar=20 μm. Msp, microspore; P, pollen; T, tapetum.

Both wild-type and *βvpe* anthers were examined to further clarify the pollen development process in the *βvpe* mutant. No obvious differences were observed between the wild type and the *βvpe* mutant from stage 7 to stage 10 (Fig. 3A–D, G–J). However, pollen development was partially abnormal and the number of mature pollen grains was significantly decreased after stage 10 in *βvpe* mutants. At stage 11, well-developed microspores were expanded and tapetal cells are almost completely degraded, with only a few remnants in wild-type anthers. In contrast, some microspores were still vacuolated and tapetal cells still remained in the *βvpe* mutant (Fig. 3E, K). At stage 12, mature pollen grains were formed and tapetal cells are completely degraded in the wild type. However, although some *βvpe* pollen grains exhibited normal development, other pollen grains were shrunken and defective (Fig. 3F, L).

### Abnormal pollen development in *βvpe* mutants

**Fig. 4.**
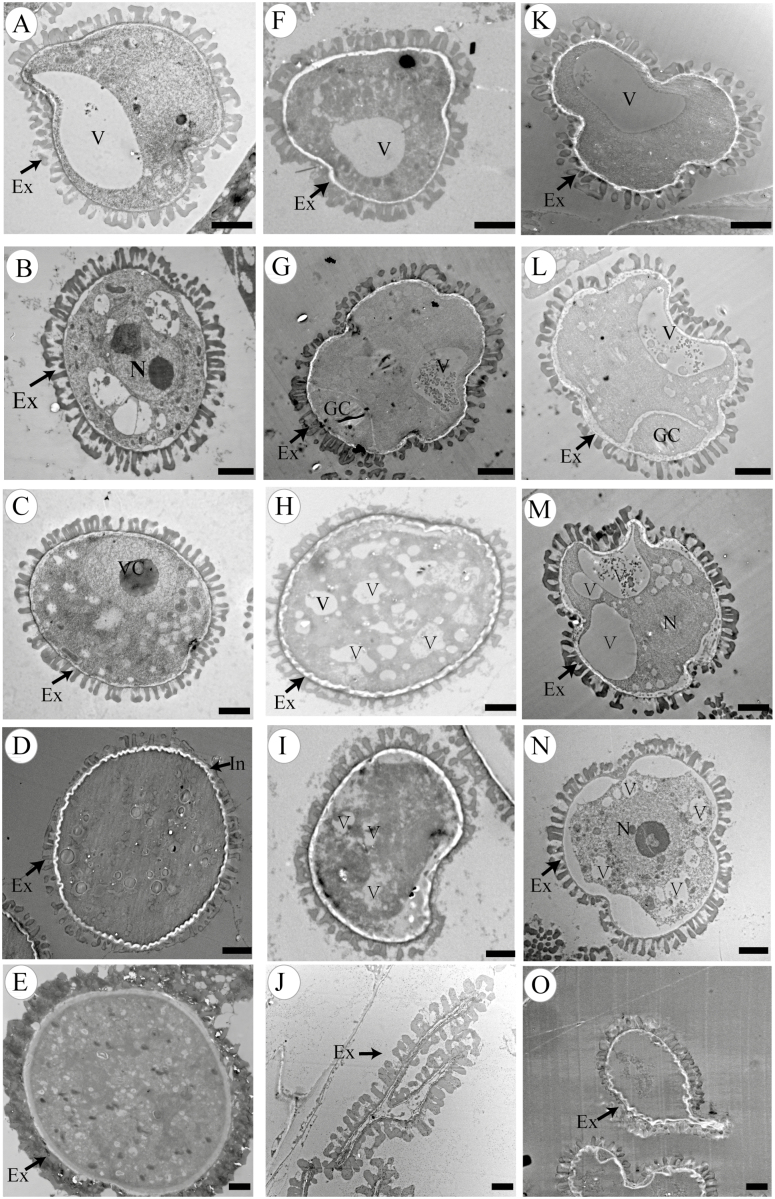
Transmission electron micrographs of microspores from the wild type and *βvpe* mutant. Microspores of different developmental stages in the wild type (A–E), *βvpe* mutant CS_1007412 (F–J), and *βvpe* mutant SAIL_50_F12 (K to O): (A), (F), and (K) stage 9; (B), (G), and (L) stage 10; (C), (H), and (M) stage 11; (D), (I), and (N) stage 12; (E), (J), and (O) stage 13. Scale bar=2 μm. Ex, exine; GC, generative cell; In, intine; N, nucleus; T, tapetal cell; V, vacuole; VC, vegetative cell.

We performed TEM to investigate pollen development in *βvpe* mutants. At stage 9, the development of the orderly microspore exine structure proceeded in both the wild type and *βvpe* mutants (Fig. 4A, F, K). However, obvious differences were observed in microspore development between *βvpe* mutants and the wild type at the beginning of stage 10. At stage 10, many oil bodies were present in the microspore, and one nucleus was present in the lenticular-shaped generative cell in the wild type. In *βvpe* mutants, unlike in the wild type, pollen cytoplasm development was incomplete, with few oil bodies and an indistinct generative cell. Some of the pollen grains were shrunken and abnormally shaped in *βvpe* mutants at this stage (Fig. 4B, G, L). At stage 11, the immature pollens grains continued to develop and the vacuole completely disappeared in the wild type (Fig. 4C). However, numerous small vacuoles were distributed throughout the cytoplasm of the *βvpe* pollen grains (Fig. 4C, H, M). At stage 12, the typical pollen wall was completely established in the wild type. In the mutants, immature pollen still contained numerous small vacuoles and some of the pollen grains were shrunken (Fig. 4D, I, N). At stage 13, wild-type pollen was fully mature. In the *βvpe* mutants, the shriveling and invagination of some of the pollen grains indicated that they were not mature in the *βvpe* mutants (Fig. 4E, J, O).

### Abnormal degradation of the tapetum in *βvpe* mutants

**Fig. 5.**
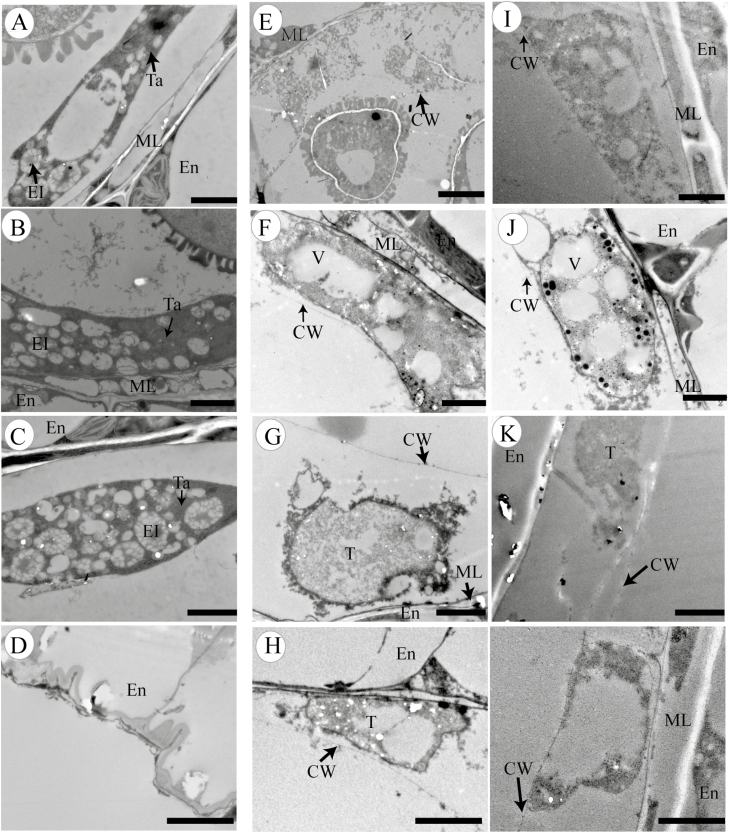
Transmission electron micrographs of anthers from the wild type and *βvpe* mutant (A–D) Wild type; (E–H) *βvpe* mutant CS_1007412; (I–L) *βvpe* mutant SAIL_50_F12: (A), (E), and (I) stage 9; (B), (F), and (J) stage 10; (C), (G), and (K) stage 11; (D), (H), and (L) stage 12. Scale bar=2 μm. CW, cell wall; El, elaioplast; En, endothecium; ML, middle layer; T, tapetal cell; Ta, tapetosome; V, vacuole.

A TEM assay was used to visualize differences in the tapetal cells between the wild type and *βvpe* mutants in stages 9–12. These results indicate that the degradation of tapetal cells in *βvpe* mutants was abnormal and that the formation of secretory organelles had greatly decreased. At stage 9 in the wild type, the tapetal cell wall had completely degraded and the tapetosome containing many lipid materials appeared in the binucleate tapetal cell (Fig. 5A). However, the tapetal cell wall partially remained in the *βvpe* mutant, indicating that the mutant’s tapetal cells had failed to transform properly into the polar secretory type and had only formed a few secretory vacuoles and vesicles (Fig. 5E, I). At stage 10, the nuclei of wild-type tapetal cells had already degraded, the numbers of tapetosomes and elaioplasts were enriched, and the tapetal cells continuously released fibrillar materials into the anther locules (Fig. 5B). In contrast, few elaioplasts and almost no tapetosomes had formed in the *βvpe* mutant, resulting in little fibrillar material being released from its tapetal cells (Fig. 5F, J). At stage 11, wild-type tapetal cells reached the end of their PCD and were filled with tapetosomes and elaioplasts (Fig. 5C). However, in the *βvpe* mutant, tapetal cell walls still remained, and, while the cytoplasm of tapetal cells had undergone degeneration, no obvious tapetosomes or elaioplasts were present (Fig. 5G, K). At stage 12, tapetal cell degeneration had finished in the wild type (Fig. 5D), but tapetal cell remnants with partially undegenerated cell walls remained in the *βvpe* mutant (Fig. 5H, L).

**Fig. 6.**
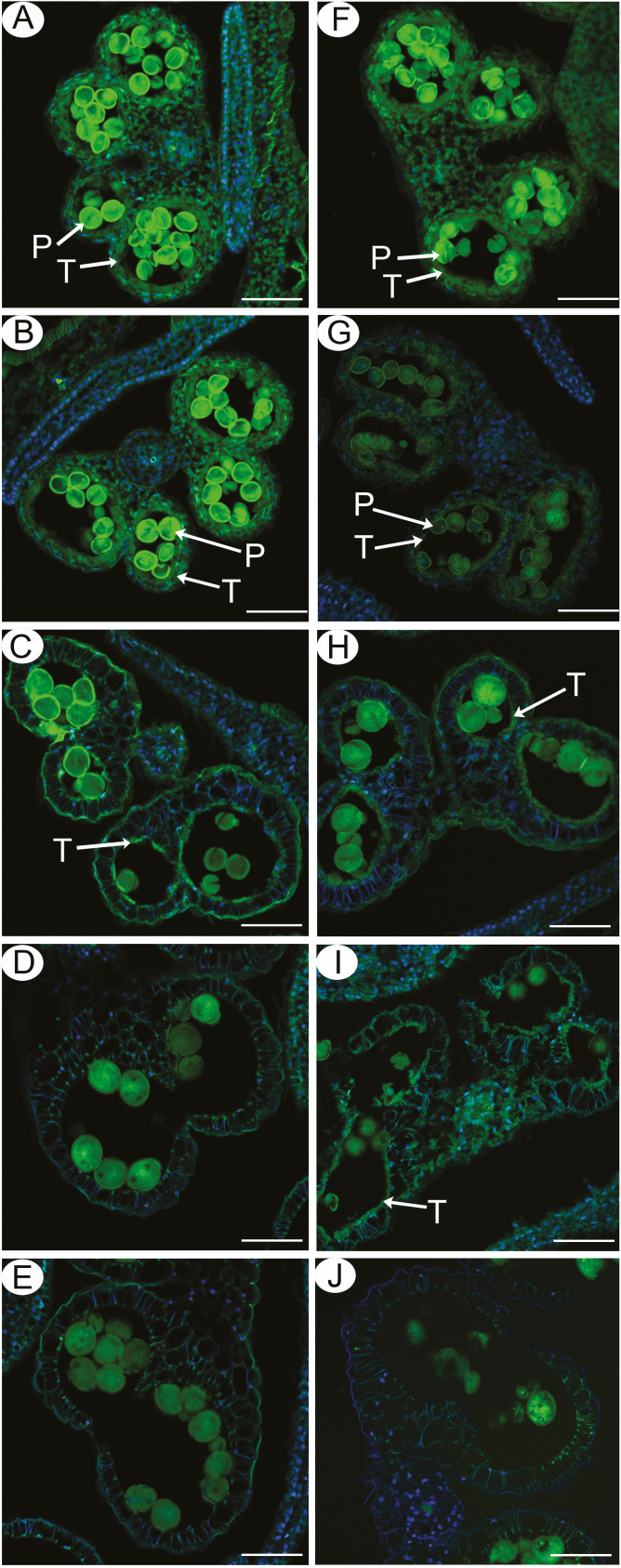
DNA fragmentation in wild-type and *βvpe* anthers. Anthers of the five developmental stages in the wild type (A–E) and the *βvpe* mutant (CS_1007412) (F–J) were compared for nuclear DNA fragmentation using the TUNEL assay. The green fluorescence (520 nm) is TUNEL-positive staining of nuclei (arrowheads). (A) and (F), stage 10; (B) and (G), early stage 11; (C) and (H), late stage 11; (D) and (I), stage 12; (E) and (J), stage 13. P, pollen; T, tapetum. Scale bars=20 μm.

To further confirm the abnormalities in *βvpe* mutant tapetal PCD, a TUNEL assay was performed in wild-type and *βvpe* mutant anthers at different developmental stages. No obvious differences in green TUNEL-positive signals were observed in tapetal PCD between *βvpe* mutants and the wild type at stage 10 (Fig. 6A, F), indicating that the starting time of tapetal cell DNA fragmentation was not retarded in *βvpe* mutants. At early stage 11, intensely green TUNEL-positive signals were present in the degenerating tapetum of the wild type (Fig. 6B), whereas TUNEL-positive signals were weaker in the *βvpe* tapetal cells at this stage (Fig. 6G). At late stage 11, intensely green TUNEL-positive signals were significantly decreased in the degenerating tapetum of the wild type (Fig. 6C), whereas TUNEL-positive signals were obvious in the *βvpe* tapetal cells at this stage (Fig. 6H). At stage 12, no TUNEL-positive signals were observed in the wild-type anther tapetum due to complete degeneration of the tapetal cells (Fig. 6D). However, TUNEL-positive signals were still present in the tapetal cell remnants of the *βvpe* mutant (Fig. 6I). At stage 13, no TUNEL-positive signals were observed in the wild-type anther tapetum (Fig. 6E) and the *βvpe* mutant (Fig. 6J). These results suggested that the tapetal cell DNA fragmentation was retarded in the *βvpe* mutant.

### Proβvpe: *βvpe* translational fusion complements the *βvpe* mutation

**Fig. 7.**
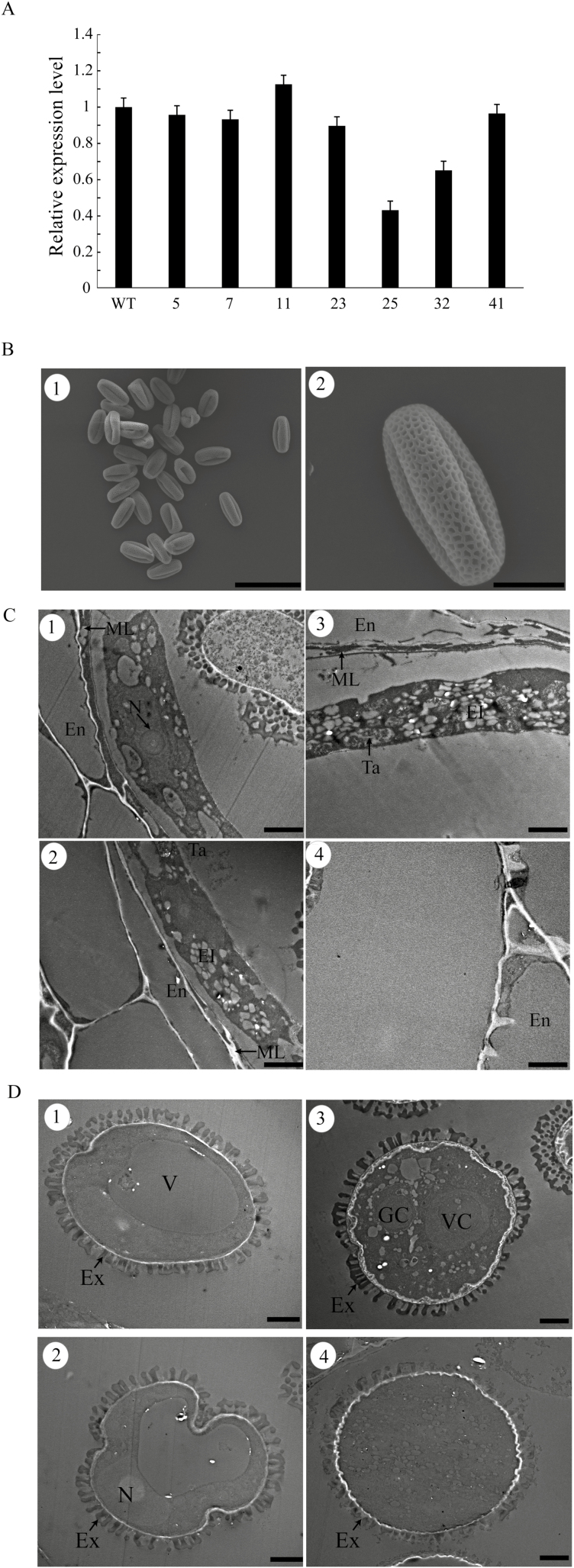
Complementation of the *βvpe* mutant (CS_1007412) by *βVPE* cDNA. (A) qRT–PCR of *βVPE* expression in bud tissues of complementation lines. (B) SEM of mature pollen grains of complementation lines: (B1) scale bar=50 μm; (B2) scale bar=10 μm. (C) Transmission electron micrographs of the tapetum development in complementation lines: scale bar=2 μm. (D) Transmission electron micrographs of microspore development in complementation lines: scale bar=2 μm.

A complementation experiment was performed to confirm that the *βvpe* mutant phenotype was attributable to the loss of *βVPE* function. An 1847 bp promoter of *βVPE* (Pro*βVPE*) and the 1713 bp *βVPE* cDNA sequence were cloned into the pCAMBIA 1300 vector and introduced into *βvpe* mutant plants (CS_1007412). *βVPE* transcript levels in 46 independent transgenic plants were detected by qRT–PCR. Among 46 independent transgenic plants, 35 plants contained similar numbers of transcripts to the wild type (Fig. 7A, lines 5, 7, 11, 23, and 41) and eight were partially restored (Fig. 7A, lines 25 and 32). The negative control lines transformed with the empty pCAMBIA 1300 vector revealed a sterile phenotype similar to that of the *βvpe* mutant.

The pollen grains of complementation lines 5, 7, and 11 were detected by SEM examination. Compared with the wild type (82.14%, 92 of 112), normal pollen grains were not significantly lower in the complementation experiment (86.36%, 95 of 110) (Fig. 7B). The restored transgenic lines displayed normal tapetal degeneration, pollen development, and fertile pollen grains. The tapetum degradation and microspore development of anthers during stages 9–12 of the restored transgenic lines (complementation lines 5, 7, and 11) were normal. The formation of tapetosomes and elaioplasts was normal and the degradation of the cell wall in tapetal cells was complete in the restored transgenic lines (Fig. 7C). The development of pollen in the restored transgenic lines was also the same as that of the wild type (Fig. 7D). These results indicate that the phenotype of the *βvpe* mutant is caused by the loss of βVPE.

### βVPE can transform the precursors CEP1, RD19A, and RD19C into mature proteins *in vivo*

**Fig. 8.**
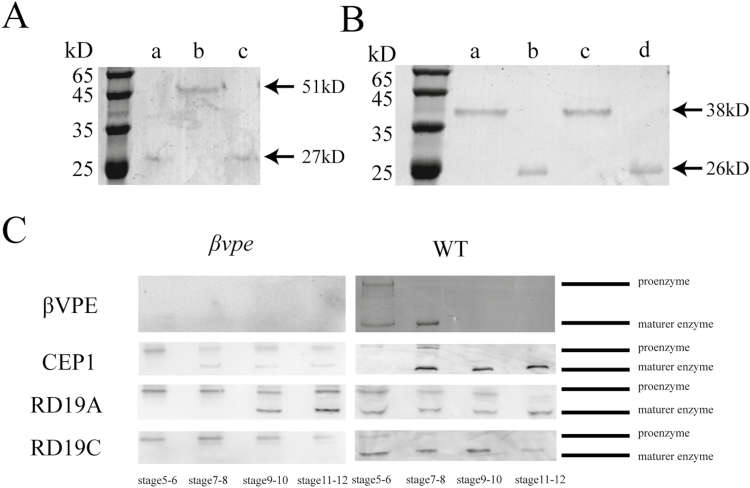
Activation of the proproteases CEP1, RD19A, and RD19C. (A) Purified and mature recombinant βVPE: (a) and (c) mature βVPE protein created by self-cleaving at pH 5.2; (b) recombinant pro-βVPE. (B) Purified and mature recombinant CEP1: (a) recombinant pro-CEP1 at pH 7.0; (b) mature CEP1 protein created by self-cleaving at pH 3.0; (c) pro-CEP1 at pH 5.2; (d) mature CEP1 protein created by mediation of βVPE. (C) Immunoblot analysis of total anther protein extracts from stages 5 to 12 with anti-mature CEP1 antibody, anti-mature RD19A antibody, and anti-mature RD19C antibody.

To further characterize the properties of βVPE, induction of the expression construct pET30a-βVPE, which encodes the full-length βVPE cDNA minus the signal peptide, was performed in *Escherichia coli*, resulting in overexpression of the recombinant protein. The molecular weight of the purified recombinant proβVPE enzyme was 51 kDa according to SDS–PAGE analysis (Fig. 8A, lanes 1 and 3). The mature protein had a mol. wt of 27 kDa and self-cleaved from the proβVPE protein at pH 5.2 (Fig. 8A, lane 2).

To further characterize whether βVPE could transform the maturation of CEP1 *in vitro*, we induced the expression construct pET30a-CEP1, which encodes the full-length CEP1 cDNA minus the signal peptide, in *E. coli*, resulting in overexpression of the recombinant protein. The molecular weight of the purified recombinant proCEP1 enzyme was 38 kDa, according to SDS–PAGE analysis, and it transformed into the 26 kDa mature protein by self-cleaving at pH 3.0 (Fig. 8B, lanes 1 and 2). The CEP1 proprotein was not able to transform into the mature protein by self-cleaving at pH 5.2 (Fig. 8B, lanes 3). When mature βVPE was added, the 26 kDa mature protein CEP1 was detected at pH 5.2 (Fig. 8B, lanes 4). These results indicate that self-cleavage at pH 3.0 or the catalysis of βVPE at pH 5.2 could transform the maturation of CEP1 *in vitro*. To determine whether βVPE could transform the inactive precursor proteases to mature proteins *in vivo*, immunoblotting was employed to investigate three cysteine proteases (CEP1, RD19A, and RD19C) from stages 5–12 in both wild-type and *βvpe* mutant anthers. In the wild type, the 38 kDa CEP1 proenzyme appeared in stages 5–6, the mature 26 kDa enzyme appeared during stages 7–8, and only the mature 26 kDa enzyme was found abundantly in the anther during stages 9–12. However, in *βvpe* mutants, the content of mature 26 kDa CEP1 was much lower, and a large amount of proenzyme still existed during stages 9–12 (Fig. 8C). In the wild type, the RD19A proenzyme was present in stages 5–10, but the 26 kDa mature enzyme did not appear until stages 7–8, and only the mature 26 kDa enzyme was found abundantly in the anther during stages 11–12. However, in the *βvpe* mutant, the content of mature RD19A enzyme was greatly diminished, the mature enzyme appeared during stages 9–10, and considerable quantities of RD19A proenzyme were still observed during stages 9–12 (Fig. 8C). In the wild type, the RD19C proenzyme was present in stages 5–6, and the mature 26 kDa mature enzyme appeared during stages 7–12. However, only the RD19C proenzyme was present during stages 5–12 in the *βvpe* mutant (Fig. 8C).

Together, these results demonstrate that βVPE is important for the transformation of the proproteases CEP1, RD19A, and RD19C into fully active mature enzymes that are necessary for proteolytic processing during anther development.

## Discussion

### βVPE should transform into its mature form by self-cleavage in response to acidification of the vacuole during anther development

Vacuolar processing enzymes are endopeptidases with substrate specificity for asparagine residues. They are synthesized as inactive larger proprotein precursors, from which the C- and N-terminal propeptides are sequentially self-catalytically removed to produce the active mature forms under acidic conditions. Previous research has shown that the 51 kDa pro-γVPE (does not contain the signal peptide) is self-catalytically converted to a 43 kDa intermediate form and then to the 40 kDa mature form at pH 5.5 (Kuroyanagi *et al.*, 2002). In Arabidopsis seeds, βVPE has a 37 kDa intermediate form and a 27 kDa mature form (Shimada *et al.*, 2003). In our research, high levels of expression of βVPE were detected in stage 5–8 Arabidopsis anthers. βVPE existed as pro-βVPE at stages 5–6 and was self-catalytically converted into the 27 kDa mature form in stages 5–8 *in vivo*. A further assay using prokaryotic expression *in vitro* revealed that pro-βVPE transformed into mature protein at pH 5.2. Previous studies have reported that vacuole acidification begins during late stage 6 and vacuole degradation is complete by late stage 8. Pro-βVPE should transform into its mature form by self-cleaving during the early stages of vacuole acidification in anther development. With vacuolar rupture complete by late stage 8, mature βVPE is released into the cytoplasm, where it is degraded. This finding suggests that βVPE acts primarily in vacuoles, not in the cytoplasm, during anther development. Tapetal development is controlled by a complex transcriptional regulatory network. Many transcription factors are involved in anther cell differentiation and tapetal development. An analysis of the known microarray data revealed that βVPE expression is down-regulated 1.3, 4.0, and 3.9-fold in *ms1*, *ems1*, and *spl* mutants, respectively, and up-regulated 3.6-fold in *tdf1* mutants (Vizcay-Barrena *et al.*, 2006; Wijeratne *et al.*, 2007; Phan *et al.*, 2011). However, *βVPE* expression is not significantly altered in the *dyt1*, *rpk2*, *ams*, *ashh2*, *mia*, *myb80*, and *roxy1roxy2* mutants, whereas other papain-like cysteine proteases, such as RD19C, RDL1, RD19A, THI1, and RD21A, show changes in expression level to varying degrees (Ito *et al.*, 2007; Yang *et al.*, 2007; Bernoux *et al.*, 2008; [Bibr CIT0025][Bibr CIT0010]; Feng *et al.*, 2012; Li *et al.*, 2017). *SPL/NZZ* and *EMS1/EXS* are expressed during anther differentiation and probably act as early regulators of *βVPE* expression around stage 5. These results suggest that βVPE is involved in the SPL/NZZ, EMS1/EXS, and TDF1 pathways regulating tapetal development and degeneration.

### βVPE directly participates in the maturation of cysteine proteases

Previous reports have shown that premature protease is transported to the protease vesicles, presumably via the endoplasmic reticulum, and then transported into the vacuole, ricinosomes, or lysosome, and transformed into mature protease in response to acidification at stage 6 by self-cleavage or protease-dependent maturation. For example, castor bean CysEP and tomato CysEP are active at pH 4–6.5 (Greenwood *et al.*, 2005; Senatore *et al.*, 2009). Previous studies in our lab have shown that the proenzyme of CEP1 is transformed into the mature form by self-hydrolysis in vacuoles at pH 3.0 during Arabidopsis anther development (Zhang *et al.*, 2014).

Our results indicate that the mature βVPE enzyme could activate pro-CEP1 *in vitro* and transform the inactive precursors CEP1, RD19A, and RD19C into mature proteins *in vivo*. The maturation of CEP1 and RD19A was greatly suppressed, and the mature form of RD19C was completely undetectable, in the *βvpe* mutant. Previous research in our lab revealed that pro-CEP1 is transformed into its mature form by self-hydrolysis at stages 6–8 with acidification of the vacuole but before vacuole rupture. In the *βvpe* mutant, however, CEP1 proenzyme was detected during stages 9–13. This finding suggests that the CEP1 proenzyme transforms in two ways: by self-hydrolysis and by the action of βVPE. Self-hydrolysis of βVPE occurs at pH 5.2 and self-hydrolysis of CEP1 occurs at pH 3.0, suggesting that the maturation time of VPE is earlier than that of CEP, and that βVPE could activate the transformation of CEP1 into a mature protein in vacuoles. The maturation of RD19A was also seriously suppressed, suggesting that βVPE processing is one way in which proRD19A is transformed into a mature protein. However, RD19A proenzyme transformed into mature RD19A enzyme after vacuole rupture without the mediation of βVPE. The mature form of RD19C was completely undetectable in the *βvpe* mutant, suggesting that the transformation of proRD19C into a mature protein can only occur through a βVPE-mediated process.

These results indicate that βVPE is involved in the maturation of cysteine proteases during tapetum development.

### βVPE is indirectly involved in pollen development and tapetal cell degradation

Many plant cysteine proteases are implicated in a variety of PCD events in various tissues, including xylogenesis (Han *et al.*, 2012), leaf and flower senescence (Chen *et al.*, 2002; Senatore *et al.*, 2009), seed development and germination (Greenwood *et al.*, 2005), and tapetum development (Zhang *et al.*, 2014). The tapetum plays an important role in microspore development, providing enzymes for the release of microspores from tetrads as well as nutrients for pollen development and essential components for the development of pollen walls. The premature termination of PCD in tapetal cells can interrupt the nutrient supply for microspore development, eventually leading to male infertility (Ku *et al.*, 2003; Varnier *et al.*, 2005). Previous research has shown that the cysteine protease CEP1 is expressed specifically in the tapetum, and that tapetal PCD is aborted and pollen fertility is decreased with abnormal pollen exine in the *cep1* mutant (Zhang *et al.*, 2014). In our study, loss of βVPE function in the *βvpe* mutant caused the failure of some CEP1 proproteins to transform into mature enzymes. A TEM analysis confirmed the failure of tapetal cell wall degeneration, a large decrease in the formation of elaioplasts and tapetosomes, and abnormal pollen development in the *βvpe* mutant in stages 9–12 of anther development. Because βVPE is degraded at stages 9–12 of anther development, it seems that βVPE participates indirectly in pollen development and tapetal cell degradation. The main reason for abnormal pollen development and failure of tapetal cell wall degeneration in the *βvpe* mutant may be due to the failure of other proproteases, such as CEP1, RD19A, and RD19C, to mature normally. Our results also showed that some CEP1 proproteases transformed to mature protein even in the absence of βVPE, probably by self-hydrolysis. This may explain the presence of some normal pollen grains (50%) in *βvpe* mutants.

In conclusion, during the early stages of anther development, βVPE triggers the activation of other proproteases, such as CEP1, RD19A, and RD19C, to transform into mature proteins. First, pro-βVPE transforms into the mature βVPE enzyme in vacuoles. Then, βVPE activates the proproteases before vacuole rupture. After vacuole rupture, the mature βVPE enzyme quickly degrades. In our research, we first discovered that βVPE can activate proproteases. We believe that the maturation of VPE plays an important role in the degradation of the tapetum and mobilization of pollen nutrients. Our findings provide valuable insights into the regulatory network determining protein levels in the tapetum and in identifying the involvement of βVPE in anther development.
